# ‘Within Ring' Concept Treatment for Displaced H-Shaped Type IVb Fragility Fractures of the Pelvis

**DOI:** 10.1155/2021/6864910

**Published:** 2021-10-15

**Authors:** Yohei Yanagisawa, Yusuke Eda, Shotaro Teruya, Hisanori Gamada, Masashi Yamazaki

**Affiliations:** ^1^Department of Emergency and Critical Care Medicine, University of Tsukuba Hospital, 2-1-1 Amakubo, Tsukuba, Ibaraki 305-8576, Japan; ^2^Department of Orthopaedic Surgery, University of Tsukuba, 1-1-1 Tennodai, Tsukuba, Ibaraki 305-8575, Japan

## Abstract

**Introduction:**

Sacroiliac rod fixation (SIRF) preserves the mobility of L5/S1 (lumber in the pelvis), as a surgical procedure for high-energy pelvic ring fractures. The concept of SIRF method without pedicle screws into L4 and L5 is called ‘within ring' concept. *Case Presentation*. We report here the clinical results of ‘within ring' concept treatment with sacroiliac rod fixation for a case of displaced H-shaped Rommens and Hofmann classification type IVb fragility fractures of the pelvis (FFP), which A 79-year-old woman had been difficult to walk due to pain that had been prolonged for more than one month since her injury. The patient was successfully treated with SIRF, no pain waking with a walking stick and returned to most social activities including living independently within 6 months of the operation.

**Conclusion:**

SIRF is useful because it can preserve the mobility in the lumbar pelvis; not including the lumbar spine in the fixation range like spino pelvic fixation is a simple, safe, and low-invasive internal fixation method for displaced H-shaped type IVb fragility fractures of the pelvis.

## 1. Introduction

In 2013, Rommens and Hofmann suggested a classification system for FFPs [1]. The classification system is based on fracture localization and the displacement in order to categorize FFPs into four major types (type I to IV) and several subtypes (a to c). Clinical and radiological criteria are routinely used to characterize FFPs and to evaluate the proper treatment; typically, they reported type IVb FFPs to account for 15.1% of the total population (37 out of 245 cases of FFP) [1]. Type IVb is a typical fracture type that requires surgical treatment in FFPs.

As a surgical method, transsacral bar osteosynthesis is recommended for minimum invasive surgery for type IVb without displacement [1–3]. On the other hand, if the fracture is displaced, spino pelvic fixation (SPF) is recommended [1–3]. SPF is a rigid fixation construct using pedicle screws into L4 and L5, but mobility of the lumbar spine is sacrificed because the L5/S1 joint (the lumbosacral junction) is firmly fixed with spinal instruments [4, 5].

In contrast to SPF, which is a fixation method that sacrifices this mobile L5/S1 joint part, Futamura et al. reported sacroiliac rod fixation (SIRF), which preserves the mobility of L5/S1 (lumber in the pelvis), as a surgical procedure for high-energy pelvic ring fractures in 2018 [6, 7]. The concept of SIRF fixation without pedicle screws into L4 and L5 is called ‘within ring' concept. We report here the clinical results of ‘within ring' concept treatment with SIRF for a case of displaced FFP4b, which had been difficult to walk due to pain that had been prolonged for more than one month since her injury.

## 2. Case Presentation

A 79-year-old woman, 145 cm in height and 37 kg in weight, presented with a pelvic fracture due after falling from a standing height when walking in her room. She was unable to walk with low back pain. The patient was moderately healthy with some comorbidities (hypertension, cerebral infarction). The paralysis of the cerebral infarction was slight, and she was able to walk independently before this injury). Her American Society of Anesthesiologists physical status classification [8] was III.

**Figure 1:**
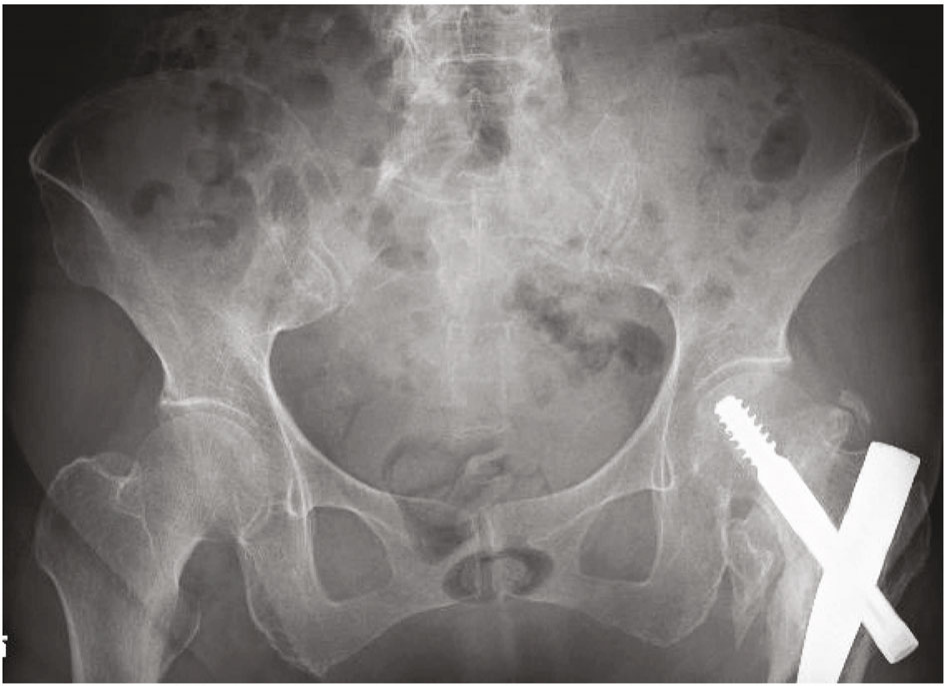
Anteroposterior pelvis radiograph.

**Figure 2:**
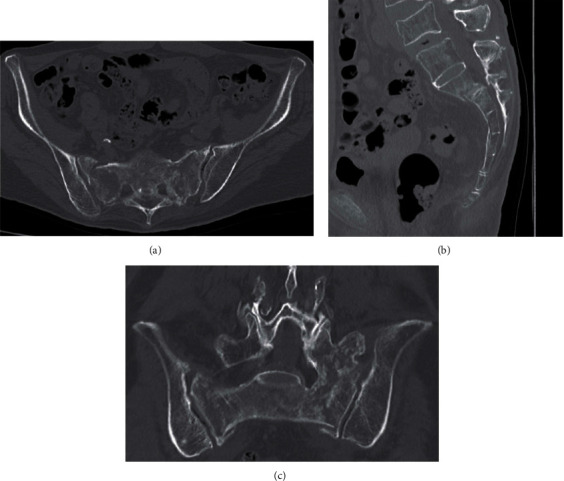
CT ((a) axial, (b) sagittal, and (c) coronal) showing displaced bilateral sacral alar fractures. L5 was bonny fused with S1.

X-ray and CT taken in the previous hospital showed few dislocations of the sacral fracture (Figure 1). After that, bed rest/conservative treatment and administration of PTH injection were performed in the previous hospital, but the pain did not improve over some weeks and walking was difficult. She was referred to our hospital because the pain persisted and the treatment was unsuccessful. CT taken at our hospital showed that the dislocation of the sacral fracture had worsened (Figure 2). The diagnosis of type IVb FFPs, displaced sacral fracture, was made from CT. In CT, a sacral corridor for inserting the transiliac transsacral (TITS) screw existed, but the TITS bar was not be approved to be used in Japan, and in this osteoporotic case with dislocation of the sacrum, there was concern about fixation with the TITS screw. On the other hand, in this case, L5 and S1 were bonny fused, and we decided to perform SIRF with inserting a pedicle screw into L5 and S1, fixing the fractured bone directly, and connecting it to the iliac screw. The operation was performed 2 months after the injury and 4 days after the transfer to our hospital under general anesthesia with the patient in the prone position.

**Figure 3:**
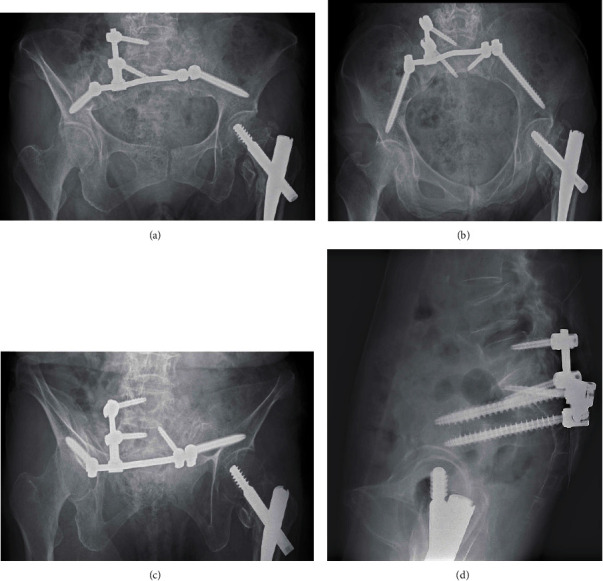
Postoperative X-ray of anteroposterior, inlet view, outlet view, and lateral view.

About 5 cm incisions were made to the skin bilaterally placed medial to both the posterior superior iliac spines (PSIS). The fascia was peeled away from the surfaces of the PSIS to develop sufficient space for manipulation of the iliac screws (IS) and S1 pedicle screws (S1PS). First, the bilateral S1PS were aimed at the promontory. The right L5 pedicle screw was inserted (left L5 pedicle screw was not inserted this time because it was inserted from the fractured site in the case). The entry point of the IS was set 2–3 cm distal to the head of the S1PS. With image intensifier position for the “teepee” view, a guidewire was placed from the PSIS toward the anterior inferior iliac spines (AIIS). Iliac screws (*φ*9.5-90 mm) were inserted [9]. Constructs were made using spinal instruments (Solera, Medtronic Co., Dublin, Ireland) (Figure 3). The surgery time was 2 h and 56 min, and intraoperative blood loss was 95 g. Immediate weight bearing as tolerated was allowed postoperatively.

**Figure 4:**
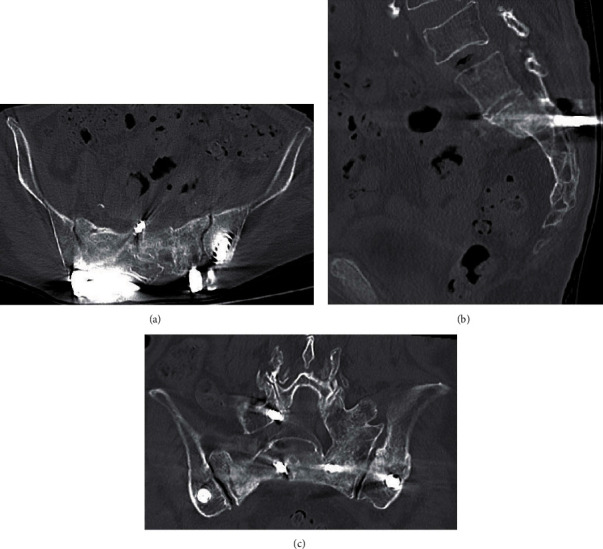
CT examinations (postoperative 6 months) ((a) axial, (b) sagittal, and (c) coronal) showing the fracture united.

The patient continued PTH injection treatment after the surgery. CT examinations (postoperative 6 months) were performed to determine the progress of bone union (Figure 4). The patient experienced no pain waking with a walking stick and returned to most social activities including living independently within 6 months of the operation. The modified Majeed score was 94 (except sexual intercourse, which was 4 points out of a possible 96) at the visit of 6 months after operation. No particular implant-related complications such as skin irritation, screw loosening, or buck out have occurred.

## 3. Discussion

SIRF is an internal fixation method for posterior pelvic ring introduced by Futamura et al. treating for high-energy pelvic ring fractures and is a method of fixation within the pelvic ring without sacrificing the mobility of L5/S1 joint. The absence of fixation of the L5/S1 joint allows conservation of mobility of the lumbar spine and resolves the concern for adjacent segment disease, screw loosing, and screw backout, etc. Since a screw is inserted into the S1 body, which is the injured region, the fracture site of S1 body can be directly fixed compared with fixation by SPF or triangular osteosynthesis [6, 7].

Transiliac internal fixation (TIF) is one fixation method using spinal instruments. TIF method is to insert one pedicle screw into each ilium and connect them with a transverse rod [1]. The concept of TIF without pedicle screws into L4 and L5 like SPF or triangular osteosynthesis is also ‘within ring' concept as SIRF. On the other hand, the clear difference between SIRF and TIF is that SIRF inserts pedicle screws into the sacral body to directly fix the fracture segment (sacral body segment). TIF does not insert pedicle screws into the sacral body. The indication for TIF is a unilateral sacral transforaminal fracture or a unilateral sacral alar fracture without dislocation; that is, the classification is FFP type II [1].

Since L5 was a bonny fused vertebra to sacrum in this case, the mobility between the pelvic ring and the lumber spine is between L4 and L5. Since screwing to L5 does not sacrifice movement in the lumbar pelvis, a pedicle screw is also inserted into the right L5 pedicle. It is possible that this L5 PS improved the fixation strength, and we should pay close attention to whether the two S1 screws and two iliac screws reported by Futamura (original SIRF construct) are sufficient for the displaced FFP type IVb, which is porotic bone.

Transsacral bar osteosynthesis is one of less invasive techniques for stabilization of the posterior pelvic ring. Rommens et al. also recommends transsacral bar osteosynthesis if possible [1, 3]. The bar is inserted through the transsacral corridor of S1. Two small incisions at the bilateral buttocks, which are in line with the transsacral corridor, are needed. A detailed examination of this case on CT images revealed that at least one could be inserted in the S1 corridor. However, the transsacral bar was not approved in Japan ministry and was not an option this time. Similarly, if it is a minor invasive surgery, the option is to insert a TITS screw. Since the TITS screw had a certain number of back outs in the osteoporotic patients comparing to transsacral bar, it was not selected because of concerns about immobility and postoperative complications [10].

SIRF is useful because it can preserve the mobility in the lumbar pelvis, not including the lumbar spine in the fixation range is a simple, safe, and low-invasive internal fixation method for displaced FFP type IVb fragility fractures of the pelvis.

## Data Availability

The datasets generated during and/or analyzed during the current study are not publicly available due to data privacy but are available from the corresponding author on reasonable request.
